# On the Use of Asbestos, in Stopping a Certain Class of Carious Teeth

**Published:** 1841

**Authors:** Solyman Brown


### ON THE USE OF ASBESTOS, IN STOPPING A CERTAIN CLASS OF CARIOUS TEETH.

[ BY SOLYMAN BROWN, M. D. ]

It is asserted by several of our professional brethren of considerable eminence, that in those cases where the cavity of a tooth extends so nearly to the nerve as to render it sensitive to the touch of an instrument, and therefore liable to be affected by the transmission of caloric through the metal composing the stopping, the use of filamentous asbestos, which very much resembles floss silk, or very fine cotton, placed at the bottom of the cavity directly over the nerve, is a valuable discovery in surgical dentistry.

The philosophy of the operation is this :—Asbestos is one of the best non-conductors of caloric known, especially among those substances which could be of use in the case in question. The conditions demanded are, 1st—That the substance employed for the purpose proposed, should be as indestructible in the tooth as the gold that covers it. 2d—That it should not be liable to absorption through the medium of the vessels of the bony structure of a tooth. 3d—That it should be as nearly as possible an absolute non-conductor of caloric.

These several indispensible pre-requisites are believed to exist in an eminent degree in fibrous and fine asbestos, a substance of which there are various qualities in different locations, and sometimes indeed in the same locality. The kind required to prevent the transmission of the two sensations of heat and cold through the metallic plug used in stopping a tooth, to the delicate nerve beneath, should be very fine in its filaments, delicately pure and white, and scarcely distinguishable from the purest untwisted or native silk. In what sections of our country it can be found in its purest state, I am not aware ; but I have been in the habit of obtaining it of an excellent quality of Mr. Chilton, of this city, well known as a distinguished chemist and apothecary.

The mode of its application is as follows :—When the cavity has been well cleaned and dried as usual, place at its bottom, or on the part adjacent to the nerve of a tooth, a sufficient quantity of the mineral made very soft and pliable, to fill about one-sixth part of the cavity when well compacted beneath the gold. Over this introduce the metallic stopping in the usual manner.

It is not claimed that a sufficient number of experiments have been already made with this substance as above described, to authorize the unqualified assertion that asbestos is destined soon to become an article of general demand among dental operators ; but enough has already been done by way of experiment, to create a strong probability that this will be the result.

The writer respectfully requests those members of the profession who may be inclined, from the momentary impulse of personal pride or vanity, to believe that what they have long been in the habit of doing is the best thing that can possibly be done in dental practice, not to reject these hints without a trial. It was thus that many of the most valuable truths of modern science, were smothered for ages beneath the dark pall of jealous superstition in centuries that have passed. It is now time that physical truths should stand or fall by the test of experiment. Theory and conjecture have sufficiently long, held their withering dominion over the fields of science. Improvements in our art, are as inevitably certain as the increase of splendour at the rising of the Sun; for the progress of all the arts and sciences, is now upward and onward. Let him, therefore, who is willing to be left behind the age to linger as one of the rear guards in the march of intellect, neglect the signs of the times, and refuse to press forward in the race of improvement. But let those who love truth for its own intrinsic beauty, and who press onward to still higher professional attainments for the sake of more extended usefulness, suffer no opportunity to pass unimproved, of augmenting the number of valuable known facts on which our science rests.

